# Comparison of molecular breeding values based on within- and across-breed training in beef cattle

**DOI:** 10.1186/1297-9686-45-30

**Published:** 2013-08-16

**Authors:** Stephen D Kachman, Matthew L Spangler, Gary L Bennett, Kathryn J Hanford, Larry A Kuehn, Warren M Snelling, R Mark Thallman, Mahdi Saatchi, Dorian J Garrick, Robert D Schnabel, Jeremy F Taylor, E John Pollak

**Affiliations:** 1 Department of Statistics, University of Nebraska, Lincoln, NE 68583, USA; 2 Department of Animal Science, University of Nebraska, Lincoln, NE 68583, USA; 3 USDA, ARS, U.S. Meat Animal Research Center, Clay Center, NE 68933, USA; 4 Department of Animal Science, Iowa State University, Ames, IA 50011, USA; 5 Division of Animal Sciences, University of Missouri, Columbia, MO 65211, USA

## Abstract

**Background:**

Although the efficacy of genomic predictors based on within-breed training looks promising, it is necessary to develop and evaluate across-breed predictors for the technology to be fully applied in the beef industry. The efficacies of genomic predictors trained in one breed and utilized to predict genetic merit in differing breeds based on simulation studies have been reported, as have the efficacies of predictors trained using data from multiple breeds to predict the genetic merit of purebreds. However, comparable studies using beef cattle field data have not been reported.

**Methods:**

Molecular breeding values for weaning and yearling weight were derived and evaluated using a database containing BovineSNP50 genotypes for 7294 animals from 13 breeds in the training set and 2277 animals from seven breeds (Angus, Red Angus, Hereford, Charolais, Gelbvieh, Limousin, and Simmental) in the evaluation set. Six single-breed and four across-breed genomic predictors were trained using pooled data from purebred animals. Molecular breeding values were evaluated using field data, including genotypes for 2227 animals and phenotypic records of animals born in 2008 or later. Accuracies of molecular breeding values were estimated based on the genetic correlation between the molecular breeding value and trait phenotype.

**Results:**

With one exception, the estimated genetic correlations of within-breed molecular breeding values with trait phenotype were greater than 0.28 when evaluated in the breed used for training. Most estimated genetic correlations for the across-breed trained molecular breeding values were moderate (> 0.30). When molecular breeding values were evaluated in breeds that were not in the training set, estimated genetic correlations clustered around zero.

**Conclusions:**

Even for closely related breeds, within- or across-breed trained molecular breeding values have limited prediction accuracy for breeds that were not in the training set. For breeds in the training set, across- and within-breed trained molecular breeding values had similar accuracies. The benefit of adding data from other breeds to a within-breed training population is the ability to produce molecular breeding values that are more robust across breeds and these can be utilized until enough training data has been accumulated to allow for a within-breed training set.

## Background

One key advantage of genomic predictors is that they can be estimated early in the life of the animal and thus allow for increased accuracy of estimated breeding values (EBV), particularly for young animals, which have not yet produced progeny. However, the benefit of the inclusion of genomic predictions into EBV estimates is proportional to the amount of genetic variation that is explained by the genomic predictor [1]. To date, in beef cattle, the American Angus Association [2], Australian Angus Association, American Hereford Association [3], American Brahman Breeders Association [4], Australian Brahman Breeders Association [5], and American Simmental Association [6,7] exploit molecular information in their National Cattle Evaluations and associations for other breeds are moving towards this goal. Although the efficacy of within-breed trained genomic predictors looks promising [3-8], it is necessary to develop and evaluate across-breed predictors for the technology to be fully applied in the beef industry. Simulation studies have reported the efficacy of genomic predictors trained in one breed and utilized to predict genetic merit in differing breeds, as well as the efficacy of predictors trained using data from multiple breeds and then used to predict the genetic merit of purebreds that were either included or excluded from training data [9-11]. De Roos and colleagues [12] showed that by combining training populations, more accurate genomic predictions could be developed, particularly when the subpopulations had not diverged for more than a few generations and for lowly heritable traits. However, research in dairy cattle has shown that when the subpopulations diverged, genomic predictors from a multi-breed training population did not have higher accuracy than predictors from single-breed training sets, except when evaluation occurred in a breed that was not represented in the training set, in which case adding multiple breeds increased the accuracy of predictors, compared to using a single-breed training set [13]. Work in other species [14] has shown that population structure can account for a substantial portion of the accuracy of genomic predictors but accounting for this structure can decrease the reliability of across-breed genomic predictors. Our objectives were to derive and evaluate genomic predictors using genotypes from the Illumina (San Diego, CA) BovineSNP50 platform for growth traits (weaning and yearling weights) in single-breed and multi-breed training data sets and evaluate them on field data.

## Methods

### Training populations

A total of three within-breed and two across-breed training populations were used:

1. A multiple-breed training population, which will be referred to as the MB population, that included five breeds (Angus, Hereford, Limousin, Red Angus, and Simmental) from a database containing 50K genotypes assembled from purebred beef cattle breeds. Animals in the database were primarily artificial insemination (AI) sires that had a substantial influence on their respective breeds and EBV with reasonably high accuracies. The only exception was the Limousin breed, for which a large number of DNA samples originated from previous DNA testing, such that only about 58% of the genotyped animals were AI sires. The MBV were trained on de-regressed EBV [15] (including weights to account for variable accuracy) for all five breeds together. Breed was fitted in the model as a fixed effect because EBV used in the training set were provided separately by each breed association, each using their own genetic base. In order to achieve a reasonable degree of independence, this training subset excluded any animal that was in the evaluation population.

2. Single-breed Angus (AN), Hereford (HH), or Limousin (LM) subsets that contributed to the MB MBV, collectively referred to as the single-breed training population (SB). The difference between MB and SB is that training was performed separately for each breed, as opposed to simultaneously for all five. The MBV trained on these single-breed training sets were computed both for subsets of the evaluation population that included the same breeds as the training set and subsets that included different breeds.

3. A multiple-breed training population consisting of AI sires from 13 breeds with high accuracy EBV and referred to as MB_2K. De-regressed EBV (adjusted for base differences and including weights to account for variable accuracy) were treated as phenotypes for training, following the methods of [15]. Due to the heterogeneity of breed-specific variance components, de-regressed breeding values and their associated weighting factors were scaled to standardize genetic variance as described by [16]. This training set represents a published across-breed prediction set from the US Meat Animal Research Center 2000 Bull Project [16], and was used for comparison with the MB MBV presented here. The MB_2K training set had 16 Limousin animals in common with the MB and Limousin SB sets. This small degree of overlap between the training sets was due to some animals having been genotyped by more than one research institution, since the data used for training the MB and SB sets did not include genotypes from the 2000 Bull Project.

The numbers of animals per breed in the SB, MB and MB_2K sets are presented in Table 1. For each trait, there were five training analyses (SB AN, SB HH, SB LM, MB, MB_2K). Each training analysis resulted in a prediction equation (a vector of estimated additive allelic effects corresponding to each SNP on the 50K chip), from which an MBV for each genotyped animal in the evaluation population could be computed. Genomic prediction equations were derived using GenSel [17], delivered via the Bioinformatics to Implement Genomic Selection (BIGS) platform (http://bigs.ansci.iastate.edu/). No pre-analysis filtering of SNPs (single nucleotide polymorphisms) based on minor allele frequency (MAF) was performed. The MB_2K predictions used a BayesCπ model [18]. The MB and SB predictions used a BayesC model with π set to 0.99 because the US beef industry (i.e., American Simmental Association and American Hereford Association) applied this approach to derive the genomic predictors that are included in National Cattle Evaluations. The de-regressed EBV for a genotyped individual was modeled as the sum of a fixed breed effect, the SNP effects times its genotype covariates, plus a random residual with variance σ^2^_e_/W, with W weights from [15]. The SNP effects had a prior distribution where a SNP effect was zero with probability π or was sampled from a normal distribution with a mean of zero and a SNP effect variance of σ^2^_g_ with probability 1-π. The SNP effect variance and the residual variance had scaled inverse Chi-squared prior distributions. Parameter π had a uniform (0,1) prior distribution in the BayesCπ model, but was assumed known in the BayesC model.

**Table 1:** Numbers of genotyped animals per breed used in the three training sets (MB, SB, MB_2K)

	**Number of genotyped animals**^**1**^		
**Breed**	**MB**	**SB**	**MB_2K**
Angus	2713	2713	373
Red Angus^2^	86	0	143
Beefmaster	0	0	63
Brahman	0	0	59
Brangus	0	0	44
Braunvieh	0	0	17
Charolais	0	0	103
Gelbvieh	0	0	113
Hereford	897	897	463
Limousin	1670	1670	104
Maine Anjou	0	0	48
Shorthorn	0	0	73
Simmental	110	0	231
Total	5476	5280	1834

^1^Animals in SB and MB were identical; only 16 Limousin animals overlapped between SB or MB and MB_2K; ^2^ given the small number of genotyped animals, Red Angus and Simmental were not used in SB.

### Evaluation population

The evaluation population consisted of genotyped animals from seven breeds in the herds of 24 seedstock producers from the Northern Plains region of the US plus any genotyped animals in their 4-generation pedigrees. The numbers of genotyped animals in the evaluation populations are summarized in Table 2. When evaluating the MB_2K MBV, animals in the evaluation population that were included in the MB_2K training set, had their MBV excluded from the analysis. Data was either extracted from existing breed association databases or using DNA samples extracted from semen or hair samples and did not require an approved animal use and care protocol.

**Table 2:** Numbers of genotyped animals per breed in the field data evaluation populations

	**Number of genotyped animals**	
**Breed**	**MB_2K**	**MB and SB**
Angus	760	962
Red Angus	50	139
Charolais	31	81
Gelbvieh	23	129
Hereford	104	185
Limousin	500	599
Simmental	38	182
Total	1506	2277

The accuracies of the various MBV trained as described above, were evaluated based on the estimated genetic correlations between each of those MBV and the corresponding phenotypes in the evaluation population. Correlations were estimated using bivariate mixed linear models in which the traits were the MBV and the corresponding phenotypic trait. The genetic correlations between the trait and MBV reflect the accuracies of MBV since the square of these correlations represents the proportion of genetic variance explained by the genomic information [1,2,6,8,16,19]. The field data from the evaluation population contained weaning and yearling weights from 48 158 and 46 429 animals born in 2008 or later, respectively, with 128 050 animals in the pedigree, of which 2277 were genotyped and therefore had MBV. The average accuracy of the EBV of genotyped animals ranged from 0.44 for yearling weight in the Limousin breed to 0.84 for weaning weight in the Charolais breed.

The model for the phenotypic trait in the bivariate model included fixed effects for contemporary group, breed composition, and heterosis, and random direct and maternal additive genetic effects. An effect of heterosis was expected in some breeds if the breed association previously had an open herd book or currently registers composite animals (i.e., American Simmental Association’s Hybrid evaluation, American Gelbvieh Association’s Balancers, North American Limousin Foundation’s LimFlex program). The heterosis effect was modeled as a direct effect using three covariates for the proportions of the animal’s British x *Bos indicus*, British x Continental, and *Bos indicus* x Continental breed composition. The model for MBV included a fixed effect for the intercept and a random direct genetic effect with the variance-covariance matrix proportional to the numerator relationship matrix. The environmental variance for MBV was fixed at 0.01% of the environmental variance of the phenotypic trait, and environmental covariances between the MBV and trait phenotype were assumed to be zero. The restricted maximum likelihood (REML) estimates of the variance components were obtained using ASReml [20]. Variance components for weaning and yearling weights were also estimated using single-trait linear mixed models based only on phenotypic data. Typical values for a collection of genetic correlations are reported based on the interquartile range that captures the middle 50% of the estimates.

## Results and discussion

Genetic parameters for weaning and yearling weights estimated using single-trait analyses of the evaluation population field data from 24 herds are presented in Table 3. In general, heritability estimates were moderate to high and within the range of the estimates reported in the literature and summarized by Koots et al. [21], although some of the estimates of direct-maternal genetic correlations are greater than expected based on literature [22].

**Table 3:** **Genetic parameters**^**1 **^**and standard errors for weaning and yearling weights per breed**

	**Weaning weight (kg)**			**Yearling weight (kg)**		
**Breed**	**h**^**2**^	**m**^**2**^	**r**_**am**_	**h**^**2**^	**m**^**2**^	**r**_**am**_
Angus	0.26±0.03	0.10±0.02	−0.43±0.10	0.27±0.04	0.05±0.02	0.27±0.22
Red Angus	0.34±0.04	0.08±0.02	0.03±0.15	0.28±0.05	0.04±0.02	0.17±0.23
Charolais	0.13±0.03	0.10±0.02	0.14±0.16	0.29±0.04	0.04±0.01	0.60±0.19
Gelbvieh	0.19±0.03	0.08±0.02	−0.17±0.12	0.18±0.04	0.06±0.02	−0.10±0.23
Hereford	0.21±0.05	0.15±0.04	−0.68±0.10	0.34±0.10	0.10±0.06	−0.27±0.25
Limousin	0.38±0.04	0.15±0.03	−0.55±0.06	0.50±0.06	0.09±0.03	−0.49±0.09
Simmental	0.30±0.04	0.10±0.03	−0.48±0.12	0.20±0.05	0.04±0.03	−0.20±0.30

^1^Genetic parameters are h^2^ = direct heritability, m^2^ = maternal heritability, r_am_ = direct-maternal genetic correlation; genetic parameters were estimated in the field data set using pedigree-based REML.

Estimates of genetic correlations between each MBV from the SB populations and its corresponding phenotypic trait are presented in Table 4. The MBV evaluated in this project generally accounted for less than 25% of the genetic variation (r_g_^2^) in weaning and yearling weights (Table 4). The estimated genetic correlations for the SB AN-trained MBV for weaning (0.36±0.07) and yearling (0.51±0.07) weights were similar to previously reported estimates i.e. ranging from 0.33 to 0.52 for weaning weight and from 0.34 to 0.64 for yearling weight [23,24]. With the exception of the SB HH-trained yearling weight MBV evaluated in Hereford, within-breed genetic correlations evaluated in the same breed as used for training were greater than 0.28, with typical values ranging from 0.36 to 0.42. However, the SB HH-trained yearling weight MBV performed poorly when evaluated in the same breed, with an estimated genetic correlation of 0.06±0.22. This may be an artifact of the fact that all Hereford field data used in evaluation were from a single herd.

**Table 4:** **Estimated genetic correlations and standard errors for within-breed trained MBV for Angus, Hereford, and Limousin**^**1**^

	**Weaning weight MBV**			**Yearling weight MBV**		
**Breed**	**Angus**	**Hereford**	**Limousin**	**Angus**	**Hereford**	**Limousin**
Angus	**0.36±0.07**	0.14±0.08	−0.06±0.08	**0.51±0.07**	0.25±0.08	−0.12±0.09
Red Angus	0.16±0.16	0.09±0.16	0.25±0.16	0.08±0.18	−0.11±0.17	0.16±0.18
Charolais	−0.17±0.19	−0.06±0.19	0.35±0.19	0.09±0.18	−0.26±0.17	0.64±0.11
Gelbvieh	0.12±0.14	0.31±0.13	−0.13±0.14	0.10±0.16	0.27±0.16	0.16±0.17
Hereford	0.04±0.21	**0.42±0.18**	0.27±0.21	0.05±0.22	**0.06±0.22**	0.23±0.22
Limousin	0.02±0.09	0.23±0.09	**0.40±0.08**	0.06±0.09	0.17±0.09	**0.28±0.08**
Simmental	−0.14±0.13	0.10±0.14	0.01±0.14	−0.11±0.17	0.06±0.18	−0.36±0.16

^1^Animals in the pedigree of the field data evaluation population bulls were excluded from training; genetic correlations and their standard errors are in bold characters when the MBV was evaluated in the breed in which it was trained.

**Figure 1:**
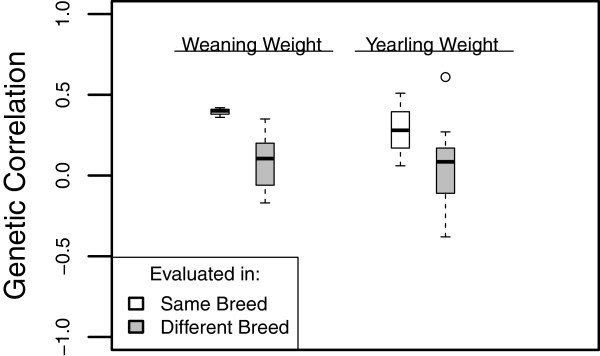
**Box plots of estimated genetic correlations between phenotypic traits and within-breed trained MBV.** MBV were evaluated either in the same breed used for training or in a different breed. Training excluded animals in the pedigrees of the field data evaluation population bulls.

Figure 1 contains box plots of the genetic correlations of SB MBV with phenotypes, evaluated either in the same breed as that used for training or in a different breed. Unlike the moderate genetic correlations obtained when the within-breed MBV were evaluated in the same breed as used for training, the genetic correlations tended to be more variable and were centered close to zero when evaluation was in a different breed. The smaller genetic correlations are consistent with the expectation that the predictive power of the MBV decreases as the genetic distance between the animals used in training and evaluation increases [10,11,25] and implies that within-breed trained MBV are of minimal value for the genetic evaluation of other breeds.

**Figure 2:**
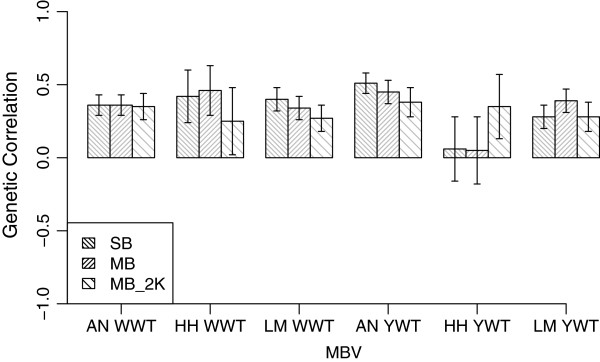
**Estimated genetic correlations and their standard errors between phenotypic traits and within-breed trained MBV.** Traits were weaning weight (WWT) and yearling weight (YWT) and were evaluated in Angus (AN), Hereford (HH), or Limousin (LM).Within-breed trained MBV were evaluated in the same breed as used in training.

Estimates of the genetic correlations between MB MBV and corresponding phenotypic traits are in Table 5. For breeds with both SB and MB MBV, the estimated genetic correlations tended to be similar (Figure 2), which suggests that either could be used with similar levels of efficacy. Considering the large contribution of AN to the training set, the estimated genetic correlations for the SB and MB trained MBV were very similar. However, based on Table 5, the use of an MB trained MBV in a breed that was not included in the training data, is not advisable.

**Table 5:** **Estimated genetic correlations and standard errors MBV, trained in two across-breed populations (MB_2K, MB**^1^**)**

	**Weaning weight MBV**		**Yearling weight MBV**	
**Breed**	**MB_2K**	**MB**	**MB_2K**	**MB**
Angus	0.35±0.09	**0.36±0.07**	0.38±0.10	**0.45±0.08**
Red Angus	−0.14±0.26	**0.33±0.14**	−0.40±0.26	**−0.03±0.17**
Charolais	0.24±0.20	0.07±0.18	0.01±0.25	−0.02±0.18
Gelbvieh	0.55±0.21	0.46±0.12	0.59±0.26	0.22±0.16
Hereford	0.25±0.23	**0.46±0.17**	0.35±0.22	**0.05±0.23**
Limousin	0.27±0.09	**0.34±0.08**	0.28±0.10	**0.39±0.08**
Simmental	0.32±0.21	**0.10±0.14**	0.68±0.23	**0.09±0.17**

^1^Estimated genetic correlations and standard errors for the MB MBV are in bold face when evaluated in breeds that were part of training.

The breeds evaluated here represent populations that have diverged over many generations, approximately 200 years since breed formation occurred. Previous work by Pryce et al. [13] using the Holstein, Jersey, and Fleckvieh breeds showed that combining divergent subpopulations in the training set does not improve the accuracy of genomic predictors over within-breed derived predictors. Pryce et al. [13] reported that the accuracy of the predictors for milk genomic breeding values in Holstein based on training in Fleckvieh was equal to 0.22 but increased to 0.42 when a second breed (Jersey) was added to the training set, which suggests that the addition of several other breeds to the training set to predict a breed that was not in the training set is beneficial. However, the same results were not consistently seen here.

Typical values for the estimated genetic correlations for the MB_2K MBV across the seven breeds ranged from 0.25 to 0.35 for weaning weight and from 0.15 to 0.50 for yearling weight. These estimates are slightly lower than previously reported for within-breed trained MBV for growth traits [11,24].

Similar to the SB trained MBV results, estimates of genetic correlations for MB trained MBV were close to zero when evaluated in a breed that was not included in the training set (Table 5). For the two breeds (Charolais and Gelbvieh) not included in the MB training set, estimated genetic correlations for Gelbvieh tended to be low to moderate. The robustness of the MB trained MBV in Gelbvieh could be due to it having closer genetic ties with breeds included in training via crossbred animals in the pedigrees. However, the average AN contribution to the Gelbvieh evaluation data was only 8.45%. When evaluated in the same breed as used for training, SB trained MBV for weight traits based on the BovineSNP50 have higher accuracy than EBV based only on pedigree and performance information.

**Figure 3:**
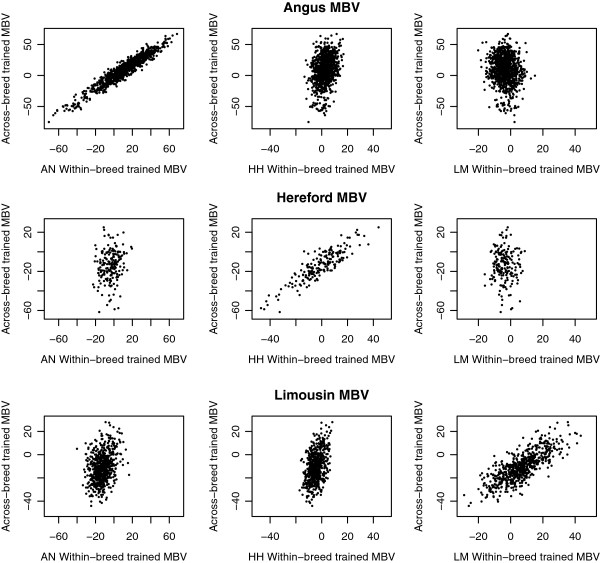
**Scatter plots of within-breed trained MBV against across-breed trained MBV for weaning weight.** Within-breed MBV trained in Angus (AN), Hereford (HH), and Limousin (LM) and evaluated in animals in the field data set of either the same or different breed.

**Figure 4:**
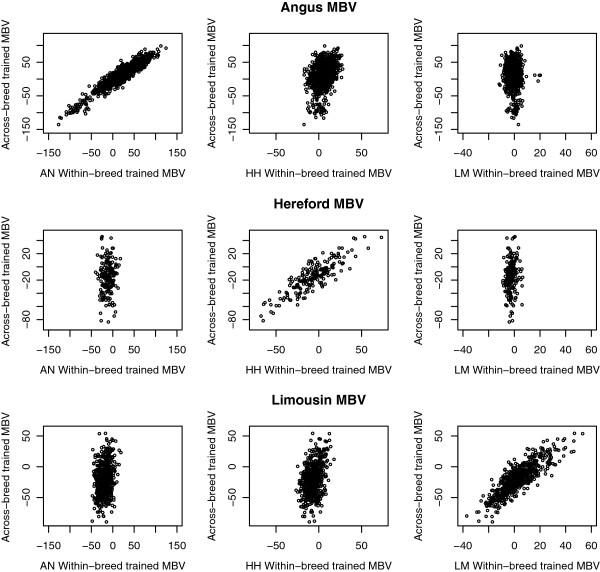
**Scatter plots of within-breed trained MBV against across-breed trained MBV for yearling weight.** Within-breed MBV trained in Angus (AN), Hereford (HH), and Limousin (LM) and evaluated in animals in the field data set of either the same or different breed.

Scatter plots of the relationship between MBV obtained from the SB and MB populations in Angus, Hereford, and Limousin breeds are presented in Figure 3 for weaning weight and in Figure 4 for yearling weight. Results show a strong linear association between the SB and MB trained MBV when applied to the same breed as used for training the SB MBV. This indicates that, on an individual breed basis, the SB and MB MBV account for much of the same variability in that breed.

There was a reduction in the proportion of genetic variance explained (r^2^_g_), and thus a reduction in the variability of MBV among animals, when SB MBV were applied to animals from a different breed (Table 4), illustrating that these MBV do not separate animals in terms of genetic merit because they do not account for a substantial portion of the genetic variance. The decrease in variability indicates that SNPs that explained variability well in the training breed did not in another breed, even in a closely related breed. This result is consistent with the finding of Gibbs et al. [2] that haplotypes longer than 250 kb are conserved across closely related breeds [26]. An alternative explanation is that some breeds may be more diverse, i.e. having a greater number of haplotypes and low frequencies of these haplotypes. Furthermore, due to selection or drift, some breeds may be fixed, or close to fixation, for certain SNPs. The greater robustness of the MB trained MBV in explaining genetic variation across breeds compared to the SB trained indicate that there are multiple collections of SNPs that can capture the underlying genetic variability and that the addition of other breeds to the training set allows the model to select the collection of SNPs that works best across multiple breeds.

The average model incorporation frequencies, i.e. the proportion of iterations of the MCMC chain with which an individual SNP enters the model, were within 0.0012 of the prior model frequency of 0.01 = 1 - π for all SNPs and for each MBV. The numbers of SNPs with model frequencies and effects that exceeded various thresholds are presented in Table 6 and Table 7. The number of SNPs in the SB trained MBV that exceeded the prior model frequency of 0.01 was greater than that in the corresponding MB trained MBV. However, within the reduced set of SNPs in the MB trained MBV that exceeded the prior model frequency threshold, there were more SNPs that had moderate to high model frequencies. While there were relatively few SNPs with large effects, the number of SNPs in the MB trained MBV with moderate to large effects was greater than in the SB trained MBV. The fact that an MB trained MBV identified a smaller set of SNPs with greater model frequencies and effects than the SB trained MBV is also consistent with the hypothesis that one consequence of adding breeds to the training set is to reduce the number of informative SNPs in the Bayes C algorithm. This decrease in the number of informative SNPs may also be due to the greater number of animals in the MB training data compared to the SB training data, resulting in a decrease in the noise associated with sampling in the MCMC algorithm.

**Table 6:** Number of SNPs with estimated model frequencies for within- and across-breed trained MBV

	**Weaning weight MBV**				**Yearling weight MBV**			
		**SB**				**SB**		
**Model frequency**	**MB**	**Angus**	**Hereford**	**Limousin**	**MB**	**Angus**	**Hereford**	**Limousin**
> 0.01	13 631	15 699	16 724	16 181	13 573	16 221	16 816	18 994
> 0.1	369	145	34	38	256	104	27	2
> 0.5	25	8	2	1	12	4	2	0
> 0.9	3	0	1	0	2	0	0	0

**Table 7:** Number of SNPs with estimated absolute effects for within- and across-breed trained MBV

	**Weaning weight MBV(kg)**				**Yearling weight MBV(kg)**			
		**SB**				**SB**		
**SNP Effect**	**MB**	**Angus**	**Hereford**	**Limousin**	**MB**	**Angus**	**Hereford**	**Limousin**
> 0.5	112	62	10	14	166	92	25	2
> 1.0	40	23	3	6	62	34	8	0
> 2.0	8	6	2	0	25	10	2	0

As suggested by [27], one benefit of adding breeds to the training set is the possibility of identifying SNPs that are in strong linkage disequilibrium with the QTL and with an allelic phase that is preserved across multiple breeds. Although this might help to identify important genomic regions harboring QTL, it is not associated with a noticeable increase in accuracy of MBV. The similar performance in individual breeds of SB and MB trained MBV supports the fact that common QTL are tracked across multiple breeds. If common QTL were not tracked, we would have expected a drop in performance when adding data from other breeds, because that would simply add noise to the data. The increase in the number of SNPs with high model frequencies and large effects in the across-breed trained MBV also supports the conclusion that common QTL are being tracked across breeds, since effects that were spread across several SNPs in each within-breed trained MBV are being assigned to a smaller set of SNPs across breeds. The MB trained MBV were, however, not consistently better than SB trained MBV.

## Conclusions

The accuracy of within- or across-breed trained MBV are substantially lower for breeds that are not included in the training set, since the estimated genetic correlations between trait MBV and their corresponding phenotypes cluster around zero. This is true even for breeds that are closely related, such as Angus and Red Angus. The addition of training data from other breeds produces an MBV where the SNP effects are concentrated onto a smaller set of informative SNPs. However, the accuracy of the across-breed trained MBV is similar to the within-breed trained MBV.

## Competing interests

The authors received no funding from The American Angus Association, Red Angus Association of America, American International Charolais Association, American Gelbvieh Association, American Hereford Association, North American Limousin Foundation, or American Simmental Association to support this study. The authors declare that they have no competing interests.

## Authors’ contributions

SDK, MLS, and KJH conceived and designed the study and assisted in the interpretation of the results. SDK analyzed the data. GLB, LAK, WMS, RMT, and EJP developed the MB_2K MBV, provided genotypes, and assisted in the interpretation of the results. MS and DJG developed and provided the genotype database and estimated the MB and SB MBV. RDS and JFT provided Angus and Limousin genotypes. SDK and MLS wrote the draft of the paper. SDK, MLS, GLB, KJH, LAK, WMS, RMT, MS, DJG, JFT, and EJP contributed to the preparation of the final manuscript. EJP directed the overall project. All authors read and approved the final manuscript.
